# Development of an International, Multicenter, Hyperbaric Oxygen Treatment Registry and Research Consortium: Protocol for Outcome Data Collection and Analysis

**DOI:** 10.2196/18857

**Published:** 2020-08-17

**Authors:** Nicole P Harlan, Judy A Ptak, Judy R Rees, Devin R Cowan, Abigail M Fellows, Judith A Kertis, Pamela M Hannigan, Janet L Peacock, Jay C Buckey

**Affiliations:** 1 Center for Hyperbaric Medicine Dartmouth-Hitchcock Medical Center Lebanon, NH United States; 2 Space Medicine Innovations Laboratory Center for Hyperbaric Medicine Geisel School of Medicine at Dartmouth Lebanon, NH United States

**Keywords:** registries, hyperbaric oxygenation, patient-reported outcome measures, registry, patient reported, outcome, measure, oxygen treatment, treatment, effectiveness, registry, health data

## Abstract

**Background:**

Hyperbaric oxygen (HBO_2_)—oxygen at pressures higher than atmospheric—is approved for 14 indications by the Undersea and Hyperbaric Medical Society. HBO_2_’s main effect is to increase oxygen content in plasma and body tissues, which can counteract hypoxia or ischemia. Laboratory studies show that HBO_2_ has effects beyond relieving hypoxia (eg, promoting angiogenesis in irradiated tissue, anti-inflammatory effects, radiosensitization of tumors, hypoxia preconditioning, and fungal growth inhibition) and has potential to treat conditions such as inflammatory bowel disease and pyoderma gangrenosum. Lack of consistently collected outcome data on a large cohort of individuals receiving HBO_2_ therapy limits its use for both established and new indications. A course of therapy often involves 30-40 visits to a hyperbaric chamber, so the number of patients seen at any given center is constrained by chamber capacity. As a result, published HBO_2_ outcome data tend to be from small case series because few patients with a particular condition are treated at a given center. To solve this problem, a registry that collects and pools data systematically from multiple institutions has been established.

**Objective:**

The aim of this study is to collect consistent outcome data across multiple hyperbaric centers to assess treatment effectiveness and establish a research consortium.

**Methods:**

A consortium of hyperbaric centers who have agreed to collect consistent outcome data on all patients seen has been assembled. Data are collected at each participating center using Research Electronic Data Capture (REDCap), a web-based, data collection system used frequently for research. Standard outcome measures have been defined for each condition, which are programmed into the REDCap data collection templates. Governance is through a consortium agreement that defines data security, data sharing, publications, liability, and other issues. Centers obtain Institutional Review Board (IRB) and ethics approval to participate, either from their own institutions or by relying on the IRB at the coordinating center at Dartmouth College. Dissemination will occur through a yearly report and by publications based on the data in the registry.

**Results:**

Early results from some common indications show significant pretreatment to posttreatment changes. Additional indications and outcome measures are being added using the procedures outlined in the consortium agreement.

**Conclusions:**

The registry collects consistent outcome information for a therapy that needs further study and a stronger evidence base. It also overcomes the challenge of collecting data from an adequate number of patients for both established and emerging indications by combining data collection from multiple centers. The data entry requirements should be within the capabilities of existing staff at any given hyperbaric center. By using REDCap, the registry can be expanded to include detailed information on particular indications and long-term follow-up on selected patients without significantly increasing the basic data entry requirements. Through the registry, a network of enrolled hyperbaric centers has been established that provides the basis for a clinical trial network.

**International Registered Report Identifier (IRRID):**

DERR1-10.2196/18857

## Introduction

Hyperbaric oxygen (HBO_2_) treatment, defined as breathing 100% oxygen at pressures greater than 1.4 atmospheres absolute, is used for 14 indications approved by the Undersea and Hyperbaric Medical Society (UHMS), such as soft tissue radiation injury and enhancement of healing in selected problem wounds (see Table 1) [1]. HBO_2_ greatly increases the amount of oxygen dissolved in plasma and is effective at relieving hypoxia. This effect is useful in conditions such as ischemic wounds or compromised flaps or grafts, where inadequate oxygenation is a factor. Laboratory studies also suggest HBO_2_ has actions beyond the relief of hypoxia (eg, promoting angiogenesis in irradiated tissue, anti-inflammatory effects, radiosensitization of tumors, hypoxia preconditioning, and fungal growth inhibition) and has potential to treat other currently unapproved conditions, such as inflammatory bowel disease and pyoderma gangrenosum [2-4]. Approximately 1350 hyperbaric chamber facilities exist in the United States, and outpatient facility claims for hyperbaric services to Medicare alone totaled US $178 million in 2015. Although HBO_2_ treatment is used for a variety of indications, much of the evidence to support its use is based on small trials, case series, and retrospective studies (see Table 1) [2,5-11].

This inconsistent evidence base has led to a range of opinions about when and how HBO_2_ should be used. For example, a common application of HBO_2_ is in treating radiation cystitis, which is supported by most insurance policies in the United States [12,13]. The evidence base and practice patterns are strong enough that when a randomized trial of HBO_2_ for radiation cystitis was attempted (ie, ClinicalTrials.gov Identifier: NCT00134628), the trial had to be closed due to poor recruitment. It was difficult to find patients who were willing to be randomized to placebo treatment and providers who were willing to refer them. In one review describing the many treatment options for radiation cystitis, the authors concluded “there are currently no adequate treatment options.” They cited HBO_2_ response rates between 27% and 92%, and recurrence rates after treatment from 8% to 63% [14]. The lower end of this response rate range suggests HBO_2_ should only be tried occasionally for selected patients, while the higher end argues that HBO_2_ should be the treatment of choice. The largest prospective study of HBO_2_ treatment for radiation cystitis included in that review was based on 40 patients [12]. Since that review, five Nordic university hospitals were able to complete a randomized trial for radiation cystitis, although they excluded from enrollment patients with severe ongoing bleeding (ie, the patients where the impact from HBO_2_ treatment would be most meaningful). The trial showed benefit from HBO_2_ [15].

This diversity of opinion and practice creates a difficult situation where the published evidence base is small, but advancing to large-scale clinical trials has been difficult. Also, hyperbaric centers worldwide are being asked to provide a stronger level of evidence to support the treatments they deliver. For example, the National Health Service (NHS) England will now support the routine use of HBO_2_ treatment only for decompression illness and gas embolism [16]. Conditions reviewed and not recommended for routine hyperbaric therapy because of the perceived lack of reliable outcome data include the UHMS-approved indications of carbon monoxide poisoning, soft tissue radiation damage, and necrotizing soft tissue infections. A new approach is needed to collect outcome data for HBO_2_ treatments.

The study of HBO_2_ treatment presents unique challenges. Because HBO_2_ treatment usually requires daily treatments over the course of 1-2 months in a hyperbaric chamber, single hyperbaric centers typically do not treat large numbers of patients and cannot accrue sufficient numbers to conduct credible studies. Furthermore, because patients are referred for treatment from other specialties, follow-up tends to be conducted by the referring specialist, and outcome data are not available to the hyperbaric program beyond the treatment period. Obtaining meaningful data on HBO_2_ outcomes requires pooling of data from multiple centers and establishing an infrastructure of centers motivated to conduct research and initiate long-term follow-up. The HBO_2_ Registry Consortium described here will provide this framework for urgently needed evaluative observational studies, with the potential to improve the clinical application of HBO_2_ dramatically. In addition, the consortium will provide an efficient platform for conducting trials on a wide range of HBO_2_ indications, as well as studies of the molecular underpinnings of the treatment itself. This consortium infrastructure could be used to develop the platform from which multiple studies could be conducted at much lower cost.

**Table 1:** Indications for hyperbaric oxygen (HBO_2_) therapy with Undersea and Hyperbaric Medical Society (UHMS) assessment of the quality and strength of evidence from the UHMS Indications report [1].

Indication^a^			UHMS assessment of evidence:quality^b^, strength^c^	Notes relevant to the registry
**UHMS-approved indications**				
	Acute thermal burn injury	A, IIa		Used only at certain centers; need to combine data from centers
	Air or gas embolism	C-LD, I		Individual centers likely to see indication only occasionally
	Carbon monoxide	A, IIa		Sporadic cases at multiple centers
	Central retinal artery occlusion	C-LD, IIb		Sporadic cases at multiple centers
	Compromised grafts and flaps	C-LD, IIa		Diverse presentations; registry good for retrospective as well as prospective analysis
	Crush injury and compartment syndrome	B-R, I		Used at some centers and not others
	Decompression sickness	C-LD, I		Use is concentrated at certain centers
	Delayed radiation injury	B-R to C-LD depending on site, I to IIb depending on site		Registry can offer consistent outcome tracking across centers
	Enhancement of healing in selected problem wounds	A, I for diabetic foot ulcers B-NR, IIb for others		Common use of HBO_2_; registry can offer consistent outcome measures needed across sites
	Gas gangrene	B-NR, I		Sporadic cases at multiple centers
	Idiopathic sudden sensorineural hearing loss	A, IIa		Used regularly at some centers and not at others
	Intracranial abscess	C-LD, IIb		Sporadic cases at multiple centers
	Necrotizing soft tissue infections	B-NR, IIa		Sporadic cases at multiple centers
	Chronic refractory osteomyelitis	B-NR, IIa to IIb depending on site		Sporadic cases at multiple centers
	Severe anemia	C-LD, IIb		Individual centers likely to see indication only occasionally
**Non-UHMS-approved indications**				
	Calciphylaxis	Not rated		No randomized controlled trials for any treatment modality; recommended as second-line therapy [5]
	COVID-19^d^	Not rated		Case series show benefit [6,7]
	Crohn disease	Not rated		Benefit seen in case reports and case series [8]
	Frostbite	Not rated		Multiple case reports show benefit; often classified as part of *acute traumatic ischemia*, like crush injury
	Otitis externa	Not rated		Case reports show benefit; Cochrane report recommends further research [9]
	Peripheral vascular disease–related ulcer	Not rated		HBO_2_ may be beneficial in selected cases
	Pneumatosis intestinalis	Not rated		Multiple case reports show benefit [10]
	Pyoderma gangrenosum	Not rated		Multiple case reports show benefit
	Raynaud syndrome	Not rated		Case reports and case series show benefit [11]
	Ulcerative colitis	Not rated		Recent randomized trial shows benefit for acute flares [2]

^a^Many of these indications are only seen episodically at any given center, so a registry is important for aggregating a sufficient number of cases to draw conclusions.

^b^Quality of evidence has five levels—Level A: highest quality, where evidence comes from more than one randomized controlled trial, a meta-analysis of high-quality randomized controlled trials, or one or more randomized controlled trials corroborated by high-quality registry studies; Level B-R: evidence comes from randomized trials; Level B-NR: evidence comes from nonrandomized trials; Level C-LD: evidence comes from limited data; and Level C-EO: evidence comes from expert opinion.

^c^Strength of evidence is classified as Class I: Strong; Class IIa: Moderate; Class IIb: Weak; Class III: No benefit; and Class III: Harm.

^d^COVID-19: coronavirus disease 2019.

Beyond the fundamental question of whether HBO_2_ treatment should be recommended for given indications, the HBO_2_ community needs data to answer more detailed questions, such as: Are some forms of radiation injury (eg, brain radionecrosis) more or less responsive than others to HBO_2_ treatment? Are some patients more likely than others to benefit from HBO_2_ for a given indication, and can those patients be identified? Having identified the patients most likely to benefit from treatment, can trials be designed more effectively to test HBO_2_ treatment (eg, crossover trials)? A well-designed registry can provide the data required to answer these questions.

## Methods

### Overview

This paper outlines the development of an international, multicenter, prospective registry consortium. A center joins the consortium by signing the consortium agreement. This agreement covers membership, governance, data sharing requirements, use of member data, publications, intellectual property, liability, confidentiality, and insurance. Data are collected using Research Electronic Data Capture (REDCap), a widely available, easily accessible, Health Insurance Portability and Accountability Act (HIPAA)-compliant, web-based data collection system [17]. Each participating center determines its own start date for data collection (ie, *reference date*) and records baseline data for every patient referred to the treatment center, whether or not treatment is indicated for, or accepted by, the patient after that start date. Patients included in the registry are those patients who have been evaluated for possible treatment of any UHMS-approved condition, or any non-UHMS-approved condition, including those who are part of research studies or trials. The registry gathers data on whether the evaluation determined that treatment was contraindicated, indicated and scheduled, or indicated but declined by the patient.

Each center’s participation is overseen by its own US Institutional Review Board (IRB) or the country-specific equivalent, or a center can opt to rely on the Dartmouth Committee for the Protection of Human Subjects (ie, the Dartmouth IRB). The REDCap-based data collection template is the same at all centers, so all centers collect the same outcome measures. Quarterly, each center performs a deidentified data download from their REDCap database to the coordinating center’s REDCap database at Dartmouth College. These deidentified data from each center are combined into a single REDCap database, which is the multicenter data registry. Optional consent for longer-term follow-up is being pilot tested at one center.

### Data Collected

The design approach to the registry is to create a system that can be used at any hyperbaric center. This means the system must be low cost and not require excessive staff effort. Although gathering data from electronic medical record systems is desirable to avoid repeated data entry, this is not practical for this project due to the diversity of medical record systems and the level of effort and funding required to standardize and update outcome measures and procedures among them. To minimize staff effort, data entry needs to be minimal, which means extensive data on comorbid conditions, medications, and medical history cannot be collected. Instead, the registry has to focus on a few key outcome measures whose data any center can collect and enter reliably. For studies using registry data where more information on the individual patients is needed, an interested investigator can obtain IRB or ethics approval to work with more detailed data at the individual centers using procedures outlined in the consortium agreement.

The registry database was initially designed by a consensus of founding members of the HBO_2_ research consortium—the Geisel School of Medicine at Dartmouth in Hanover, New Hampshire, and the Dartmouth-Hitchcock Medical Center in Lebanon, New Hampshire—and core data are collected for UHMS-approved and some nonapproved conditions. Data are collected using four main data collection instruments (see Table 2). The Demographics instrument collects demographic information as well as information on insurance and distance traveled. The Pre Treatment Information instrument collects information on the condition being treated, treatments prescribed and administered, and subjective and objective measurements of the patient’s status at treatment start. A quality-of-life measure—the EuroQol, 5-dimension, 5-level (EQ-5D-5L)—is also administered at baseline on all patients [18,19] as part of the Pre Treatment Information instrument. The Pre Surgical Information instrument collects subjective and objective measures of the patient’s status prior to a surgical intervention (eg, tooth extraction) if one is performed. The Treatment and Outcomes instrument repeats information collection of the Pre Treatment Information outcome measures, records the actual treatment given, asks about complications, and repeats the quality-of-life questionnaire.

Both indication-specific outcomes and general outcomes are collected, including HBO_2_ treatment complications (eg, changes in refraction, seizures, pneumothorax, confinement anxiety, barotrauma, and placement of pressure-equalization tubes in the ear). Wherever possible, common objective outcome measures are used. The registry uses validated questionnaires that have supporting evidence in the literature (eg, the Common Terminology Criteria for Adverse Events [CTCAE] hematuria grading scale for radiation cystitis and the European Organisation for Research and Treatment of Cancer [EORTC] Quality of Life Questionnaire Head and Neck [QLQ-H&N35] for head and neck symptoms) [20,21] as well as some questionnaires that were custom developed for the registry (see Table 2) [22-24]. Factors that might affect the effectiveness of the therapy are also collected (eg, diabetes, smoking, and other nicotine use). Future plans, including long-term follow-up and linkage to cancer registries and vital status data, depend on future funding. Table 3 lists the parameters measured [25,26].

**Table 2:** Data collection instruments and questionnaires used in the registry. Patient-reported outcomes are used in the registry whenever possible.

Instruments and questionnaires		Details^a^
**Data collection instruments**		
	Demographics	Age, race, ethnicity, biological sex, insurance, and driving distance; personal health information is only kept at individual centers
	Pre Treatment Information	Referral reason, urgency, diabetes, smoking, nicotine, indication, baseline questionnaires and outcome information, EQ-5D-5L^b^, and prescribed treatment
	Pre Surgical Information	Outcome measures prior to intervention if one is performed (eg, tooth extraction, mandibular reconstruction, etc)
	Treatment and Outcomes	Treatment given, complications experienced, and outcome measures
**Questionnaires used in the registry**		
	Bladder Questionnaire (radiation cystitis)	Seven questions from the Urinary Distress Inventory 6 plus one custom registry question on urinary bleeding [24]
	Bowel Symptoms Questionnaire	Nine questions custom developed for the registry
	Head and Neck Questionnaire	37 questions selected from the EORTC^c^ QLQ-H&N35^d^ [23] and the GRIX^e^ questionnaire [22]
	Laryngeal Soft Tissue Radionecrosis Questionnaire	Two questions based on the Chandler [20] and RTOG^f^ scales [21]
	Perianal Crohn’s Symptom Index	11 questions custom developed for the registry

^a^Details of data collection instruments include the types of data collected; details of questionnaires used in the study; and the sources used to develop the questionnaires.

^b^EQ-5D-5L: EuroQol, 5-dimension, 5-level.

^c^EORTC: European Organisation for Research and Treatment of Cancer.

^d^QLQ-H&N35: Quality of Life Questionnaire Head and Neck.

^e^GRIX: Groningen Radiotherapy-Induced Xerostomia.

^f^RTOG: Radiation Therapy Oncology Group.

**Table 3:** Indications for hyperbaric oxygen (HBO_2_) treatment and outcomes measured for the registry.

Condition			Pre- and posttreatment outcome measurements^a^
**Undersea and Hyperbaric Medical Society (UHMS)-approved conditions**			
	Acute ischemia (not crush injury or compartment syndrome)		Assessment of cyanosis in affected areas pre- and posttreatment and amputations posttreatment
	Acute thermal burn injury		Number of wounds and wound measurements
	Air or gas embolism		Glasgow Coma Scale score for brain events, troponin for cardiac events, and six-level outcome measure for all
	Carbon monoxide		Narrative on treatment and outcome
	Central retinal artery occlusion		Visual acuity (right and left)
	Compromised grafts and flaps		Graft and flap assessment (necrosis and color), number of wounds, and wound measurements
	Crush injury and compartment syndrome		Location, number of wounds, and wound measurements
	Decompression sickness		Six-level patient outcome measure
	**Delayed radiation injury**		
		Brain	Nine-Hole Peg Test and Trail-Making Test
		Larynx	Laryngeal Soft Tissue Radionecrosis Questionnaire and Head and Neck Questionnaire
		Bladder	Hematuria grade and Bladder Questionnaire
		Bowel	Bowel Questionnaire
		Jaw	Exposed bone percentage coverage if exposed bone present, osteoradionecrosis grade [25], tooth complications after extraction, and Head and Neck Questionnaire
	Enhancement of healing in selected problem wounds		Number of wounds, wound measurements, Wagner grade, and Strauss score [26]
	Gas gangrene		White blood cell count, number of wounds, wound measurements, and number of surgical interventions
	Idiopathic sudden sensorineural hearing loss		Four-frequency pure-tone average and word recognition score
	Intracranial abscess		Number of surgical interventions
	Necrotizing soft tissue infections		White blood cell count, number of wounds, wound measurements, and number of surgical interventions
	Chronic refractory osteomyelitis		White blood cell count, C-reactive protein, and number of surgical interventions
	Severe anemia		Hemoglobin; markers of end-organ damage; and narrative on treatment, complications, and outcome
**Conditions not currently UHMS approved**			
	Calciphylaxis		Location, number of wounds, wound measurements, number of surgical interventions, and subjective assessment at end of treatment
	COVID-19^b^		Pulse oximetry pre- and posttreatment, respiratory rate pre- and posttreatment, and pretreatment oxygen
	Crohn disease		Perianal Crohn’s Symptom Index and Bowel Questionnaire
	Frostbite		Number of wounds, wound measurements, and number of surgical interventions
	Malignant otitis externa		Narrative on treatment and outcome
	Peripheral vascular disease–related ulcer		Number of wounds, wound measurements, and number of surgical interventions
	Pneumatosis intestinalis		Narrative on treatment and outcome
	Pyoderma gangrenosum		Number of wounds, wound measurements, and number of surgical interventions
	Raynaud syndrome		Number of wounds, wound measurements, and number of surgical interventions
	Ulcerative colitis		Bowel Questionnaire

^a^All patients will complete a quality-of-life questionnaire—the EuroQol, 5-dimension, 5-level (EQ-5D-5L) [19]—at the start and end of treatment. Text entries are available for all indications to provide more detail about the cases.

^b^COVID-19: coronavirus disease 2019.

### Governance

The HBO_2_ Registry Consortium includes member institutions fulfilling the requirements as shown in Textbox 1. A steering committee is responsible for the governance of the registry. The Geisel School of Medicine at Dartmouth and the Dartmouth-Hitchcock Medical Center are the founding members of the consortium and each have representatives on the steering committee. The steering committee also includes a representative from each participating center. By a majority vote, with affirmative votes from the founding members, the steering committee has the authority to set the strategic direction for the registry, admit or remove members, set membership fees, establish policies, and relocate the registry. Member-initiated research protocols are approved by a majority vote of the steering committee. The committee also votes on any changes to the REDCap data collection instruments. The steering committee meets annually at the UHMS annual meeting, where changes and modifications to the registry are discussed and voted upon. Template IRB application materials are freely shared between participating centers.

**Requirements and activities of participating centers of the HBO_2_ Registry Consortium.**

Center characteristic:

- Hyperbaric oxygen (HBO_2_) treatment program

Administrative requirements:

- Ethics Committee approval, either by relying on Dartmouth or at own center
- Execution of consortium agreement, which includes agreement to share deidentified data with coordinating center and willingness to have data used for registry purposes
- Installation of, or access to, Research Electronic Data Capture (REDCap) database at the center or establishment of procedures and authorization to enter patient data securely into REDCap database hosted by another participating center

Data collection activities:

- Patient informed consent or waived consent, depending on Ethics Committee requirement
- Prospective data entry for all patients evaluated at the center; if consent is not waived, need to have 95% or greater participation over a year
- Administration of indication-specific questionnaires and/or collection of outcome measurement data before and after treatment
- Completion of annual audit and quality assurance processes; responsiveness to feedback on data quality and attainment of defined minimum data-quality standards
- Quarterly submission of deidentified patient data to coordinating center at Dartmouth College
- Option to participate in research studies, grant writing, or fundraising efforts

### Statistical Analysis

Descriptive analyses will be conducted to describe the baseline characteristics and outcomes of patients with each indication, overall and by center, including the numbers, proportions, and confidence intervals for the following: patients with each indication for treatment; those evaluated who were not treated or who were partially treated and the reasons why; baseline characteristics, including referral information and baseline outcome measures; treatments given; occurrence of each type of side effect; and outcome measures at the end of treatment with change scores and relative change scores, as appropriate. For measures with numerical scores, we will assess absolute and relative changes; for other measures, we will report outcomes in terms of the proportions with symptom resolution, improvement, no change, or deterioration. Factors associated with key outcomes will be explored using multifactorial analyses where sample size is sufficient for models to be stable. All results will be presented and discussed in terms of estimates and 95% confidence intervals to avoid focusing on *P* values alone.

## Results

**Figure 1:**
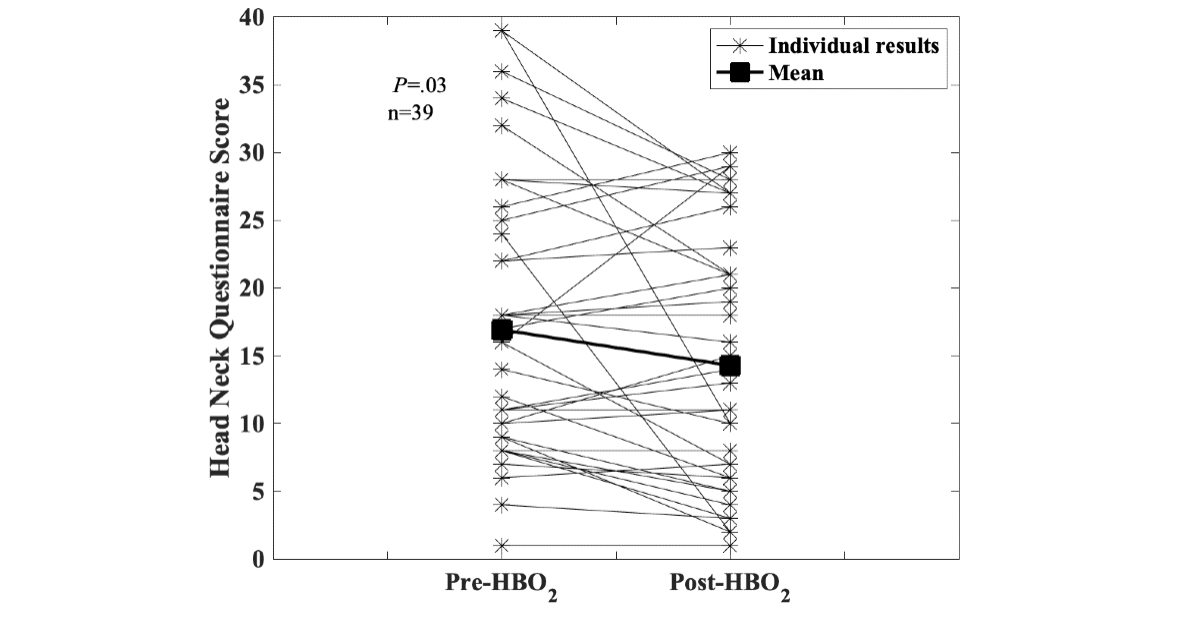
Delayed radiation injury. Scores on the Head and Neck Questionnaire before and after hyperbaric oxygen (HBO_2_) are shown: lower scores indicate fewer symptoms. This questionnaire is administered to any patient who had experienced head and neck radiation and is being treated for radiation injury in the head and neck region. Although responses vary between patients, results show lower scores posttreatment (16.9 pretreatment to 14.3 posttreatment, *P*=.03, Wilcoxon signed-rank test). By identifying patients who did not respond or who worsened, these data can guide further analyses.

**Figure 2:**
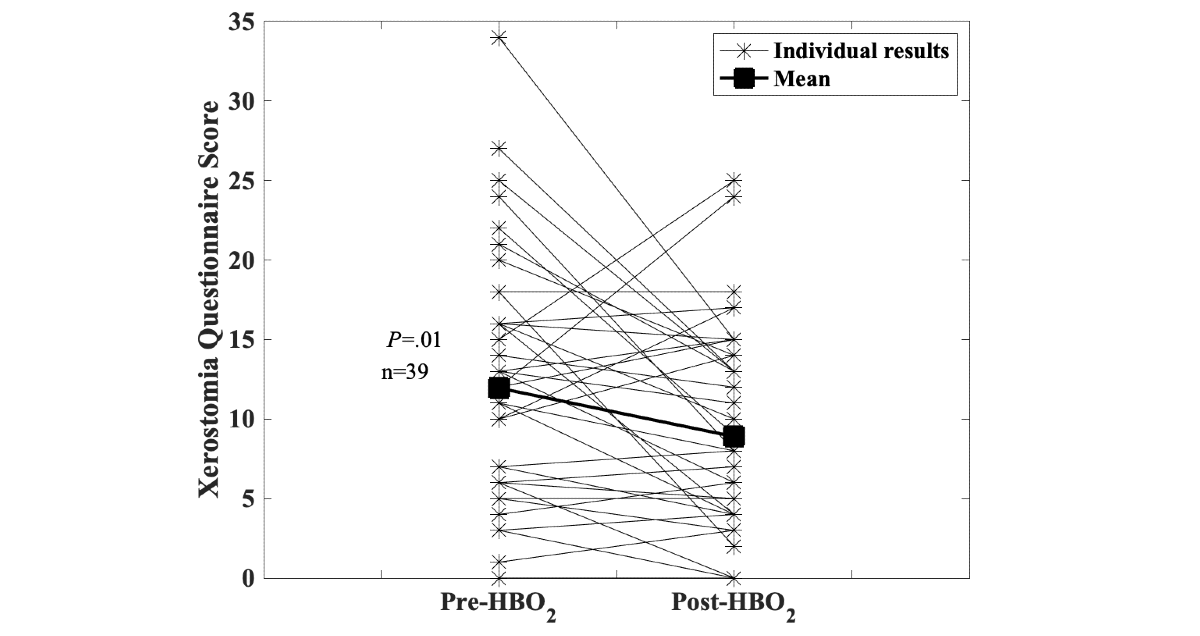
Delayed radiation injury. Scores on the xerostomia (ie, dry mouth) questions within the Head and Neck Questionnaire are shown: lower scores indicate fewer symptoms. Dry mouth is a common complication of head and neck radiation, and whether hyperbaric oxygen (HBO_2_) helps with this symptom is an open question. Early results from the registry suggest improvement (11.9 pretreatment to 8.9 posttreatment, *P*=.01, Wilcoxon signed-rank test).

**Figure 3:**
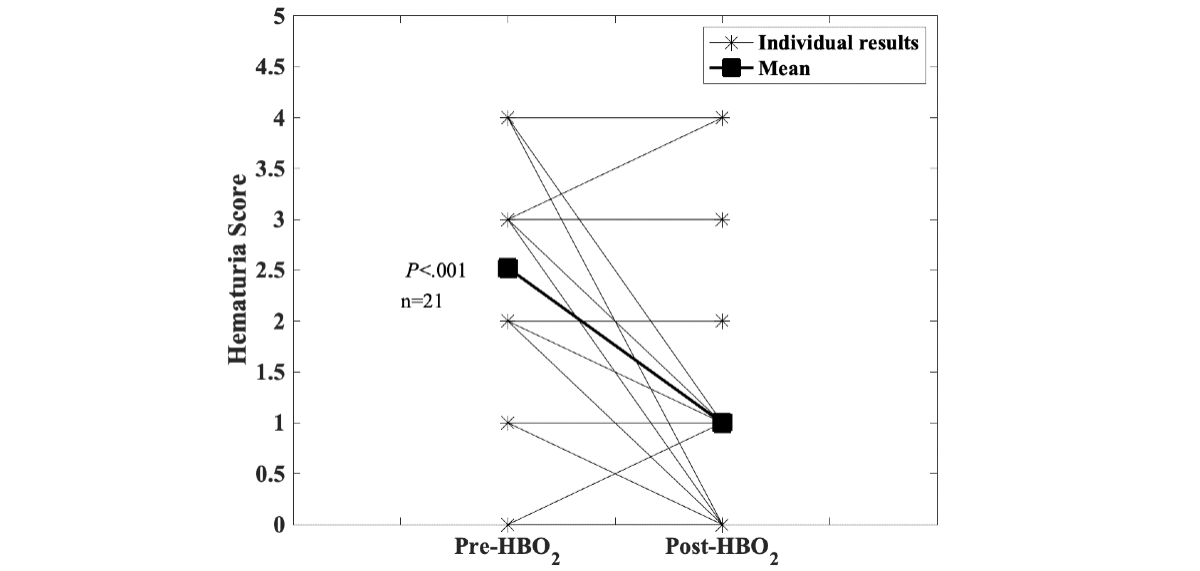
Delayed radiation injury, radiation cystitis. Scores on the hematuria scale before and after hyperbaric oxygen (HBO_2_) treatment are shown: 0=no hematuria, 1=microscopic hematuria, 2=occasional macroscopic hematuria, 3=frequent macroscopic hematuria, and 4=severe hemorrhagic cystitis. Most patients see an improvement in hematuria score (2.5 pretreatment to 1.0 posttreatment, *P*<.001, Wilcoxon signed-rank test). As the number of entries in the registry grows, these data may be useful for assessing the number of treatments needed for successful outcomes.

**Figure 4:**
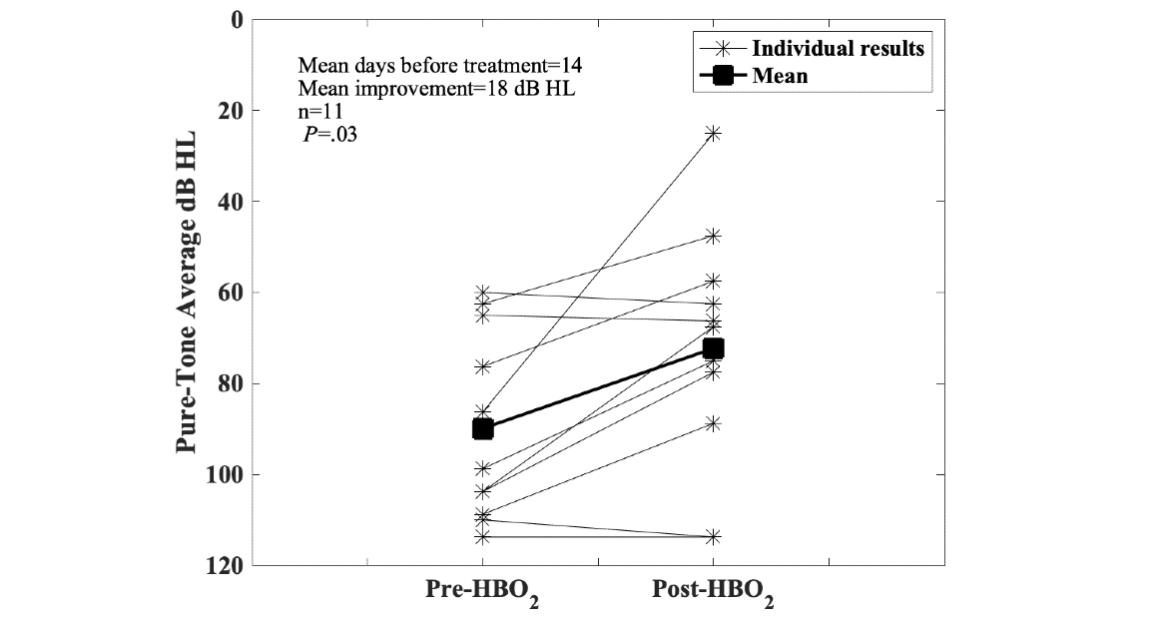
Idiopathic sudden sensorineural hearing loss. Four-frequency pure-tone averages on audiometry before and after hyperbaric oxygen treatment (HBO_2_) are shown: a lower number indicates an improvement in hearing. Most patients are experiencing an improvement in audiometric thresholds (89.9 dB hearing loss [HL] pretreatment to 72.3 dB HL posttreatment, *P*=.03, Wilcoxon signed-rank test). As the number of cases in the registry grows, these data could be used to assess how long after the hearing loss HBO_2_ may be useful.

Data collection started within the registry at the Dartmouth-Hitchcock Medical Center as a pilot project in 2012. During this time, the consortium agreement was developed and processes were established to enroll other centers. In 2019, four additional centers began entering patient data. Currently, the registry has 919 individual patient entries from four centers, with the majority from the Dartmouth-Hitchcock Medical Center (n=621). Over time, the registry is gradually collecting sufficient data to robustly explore changes before and after HBO_2_ treatment. Figure 1, for example, shows the results for the Head and Neck Questionnaire before and after HBO_2_ treatment. Figure 2 shows changes in patient-reported symptoms of xerostomia (ie, dry mouth) after receiving HBO_2_ treatment. These responses are important because whether HBO_2_ treatment has an effect on xerostomia has been a longstanding question in the HBO_2_ field [27]. Figure 3 shows changes in the hematuria score for those patients undergoing treatment with HBO_2_ for radiation cystitis. Figure 4 shows the experience to date for patients treated for idiopathic sudden sensorineural hearing loss. These preliminary results show that the registry is useful for tracking trends in outcomes and patient-reported symptoms after HBO_2_ treatment. As further patient experiences are collected, these data will also be used in retrospective analyses to determine the characteristics of those patients who responded well and of those who did not.

## Discussion

### Principal Findings

An HBO_2_ treatment outcomes registry is feasible and can provide consistently recorded outcomes from HBO_2_ treatments at multiple centers. Initial analyses of some of the outcome data are already showing significant changes after HBO_2_ treatment.

### Strengths of the Registry

A key strength of the registry is its use of REDCap [17], a free, secure, web-based, data collection system used to build and manage online surveys and databases in more than 3964 centers worldwide. The HBO_2_ REDCap database has been pilot tested and revised at the coordinating center for 6 years, and core variables have been collected on UHMS-approved indications for HBO_2_ treatment, as well as some non-UHMS-approved indications. The database includes 1996 variables, many of which are disease specific and are programmed to appear only for particular indications or situations (ie, during data entry, a staff member will only be entering data on a small subset of the variables available). Data entry takes an average of 15 minutes per case, distributed over several clinical visits. The template for the database can be exported to Excel, emailed to another center, and easily uploaded to create an identical registry that is ready for data entry. REDCap provides the ability to perform a deidentified data export from each participating center. This is sent to a central, pooled REDCap database at the Geisel School of Medicine at Dartmouth. Because of the simplicity of the software and the relatively low burden of data entry, the registry is relatively inexpensive for new centers to install and maintain. Because only deidentified data are pooled, the risk to participants is minimal.

### Limitations of the Registry

At present, the registry is following individuals who receive HBO_2_ treatment, and a registry of similar patients with similar conditions who are not receiving HBO_2_ treatment does not exist. Therefore, there is no way to compare outcomes from HBO_2_ treatment directly with outcomes from similar patients who did not receive the treatment. As the registry grows, an additional limitation will be funding, which will be needed to maintain a system of data-quality oversight, analysis, longer-term follow-up, and other registry-related activities.

### Conclusions

An outcome-focused registry for HBO_2_ treatment is needed urgently to provide both patients and providers with the information they need to decide whether and how to use HBO_2_ treatment. To be successful, this registry must be practical, easy to use, and easily expandable. The registry described here meets these requirements. The data entry requirements are not excessive and should be within the capabilities of existing staff at any given hyperbaric center. Also, by using REDCap, the registry can be expanded to include detailed information on particular indications and long-term follow-up on selected patients without significantly increasing the basic data entry requirements.

## Appendix

Multimedia Appendix 1 Presentation of the study.

## Abbreviations

CTCAE: Common Terminology Criteria for Adverse Events
EORTC: European Organisation for Research and Treatment of Cancer
EQ-5D-5L: EuroQol, 5-dimension, 5-level
HBO_2_: hyperbaric oxygen
HIPAA: Health Insurance Portability and Accountability Act
IRB: Institutional Review Board
NHS: National Health Service
QLQ-H&N35: Quality of Life Questionnaire Head and Neck
REDCap: Research Electronic Data Capture
SEAM: Scholarship Enhancement in Academic Medicine
UHMS: Undersea and Hyperbaric Medical Society
