# Supplementary material for: Development of an International, Multicenter, Hyperbaric Oxygen Treatment Registry and Research Consortium: Protocol for Outcome Data Collection and Analysis
Source: JMIR Res Protoc. 2020 Aug 17;9(8):e18857. doi: 10.2196/18857 (PMC7459436; doi:10.2196/18857)
Supplement: Multimedia Appendix 1 [file resprot_v9i8e18857_app1.pptx]

## Slide 1
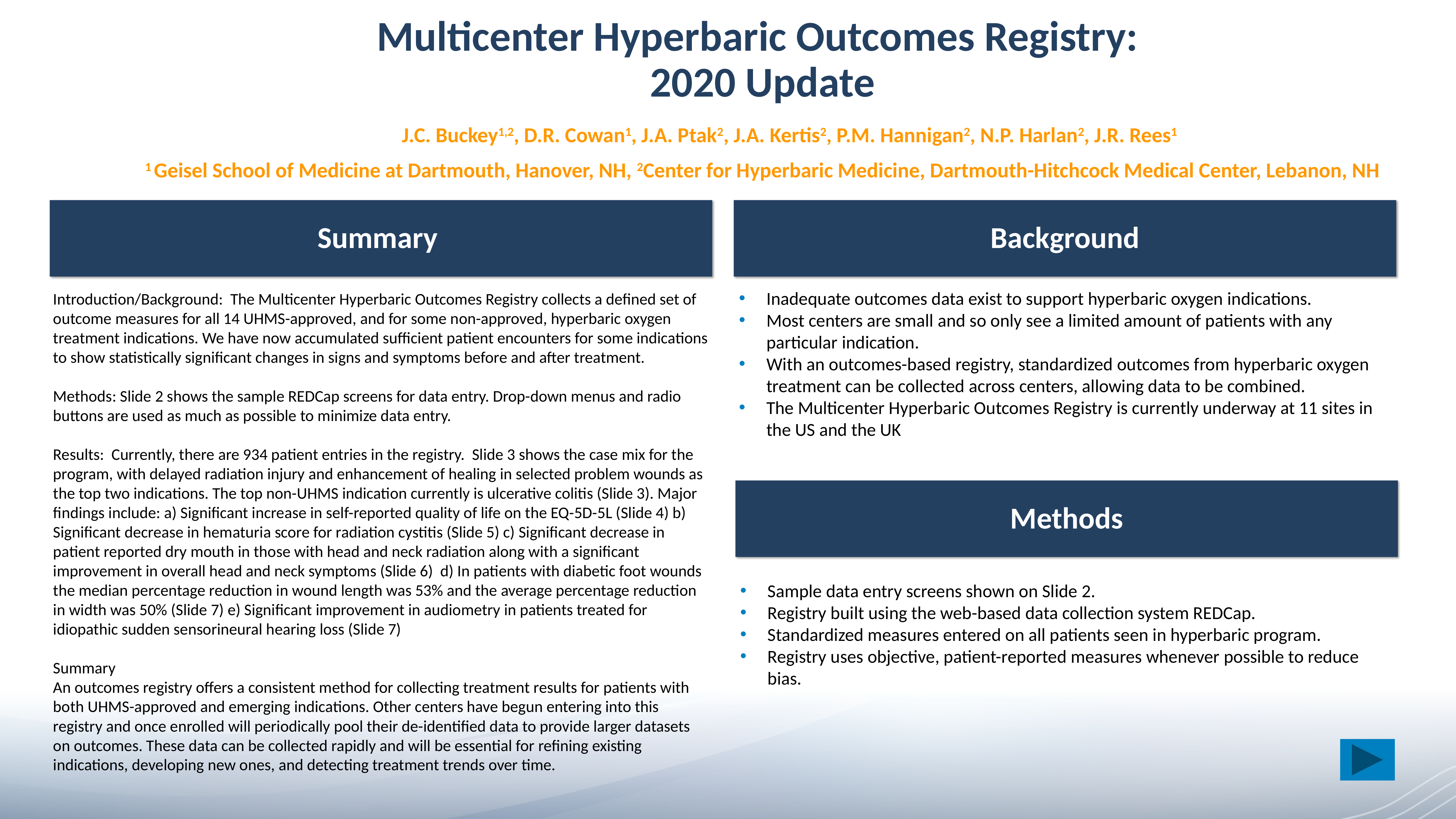

# Multicenter Hyperbaric Outcomes Registry: 2020 Update
J.C. Buckey1,2, D.R. Cowan1, J.A. Ptak2, J.A. Kertis2, P.M. Hannigan2, N.P. Harlan2, J.R. Rees1
1 Geisel School of Medicine at Dartmouth, Hanover, NH, 2Center for Hyperbaric Medicine, Dartmouth-Hitchcock Medical Center, Lebanon, NH
Summary
Background
Inadequate outcomes data exist to support hyperbaric oxygen indications.
Most centers are small and so only see a limited amount of patients with any particular indication.
With an outcomes-based registry, standardized outcomes from hyperbaric oxygen treatment can be collected across centers, allowing data to be combined.
The Multicenter Hyperbaric Outcomes Registry is currently underway at 11 sites in the US and the UK
Introduction/Background: The Multicenter Hyperbaric Outcomes Registry collects a defined set of outcome measures for all 14 UHMS-approved, and for some non-approved, hyperbaric oxygen treatment indications. We have now accumulated sufficient patient encounters for some indications to show statistically significant changes in signs and symptoms before and after treatment.
Methods: Slide 2 shows the sample REDCap screens for data entry. Drop-down menus and radio buttons are used as much as possible to minimize data entry.
Results: Currently, there are 934 patient entries in the registry. Slide 3 shows the case mix for the program, with delayed radiation injury and enhancement of healing in selected problem wounds as the top two indications. The top non-UHMS indication currently is ulcerative colitis (Slide 3). Major findings include: a) Significant increase in self-reported quality of life on the EQ-5D-5L (Slide 4) b) Significant decrease in hematuria score for radiation cystitis (Slide 5) c) Significant decrease in patient reported dry mouth in those with head and neck radiation along with a significant improvement in overall head and neck symptoms (Slide 6) d) In patients with diabetic foot wounds the median percentage reduction in wound length was 53% and the average percentage reduction in width was 50% (Slide 7) e) Significant improvement in audiometry in patients treated for idiopathic sudden sensorineural hearing loss (Slide 7)
Summary
An outcomes registry offers a consistent method for collecting treatment results for patients with both UHMS-approved and emerging indications. Other centers have begun entering into this registry and once enrolled will periodically pool their de-identified data to provide larger datasets on outcomes. These data can be collected rapidly and will be essential for refining existing indications, developing new ones, and detecting treatment trends over time.
Methods
Sample data entry screens shown on Slide 2.
Registry built using the web-based data collection system REDCap.
Standardized measures entered on all patients seen in hyperbaric program.
Registry uses objective, patient-reported measures whenever possible to reduce bias.

## Slide 2
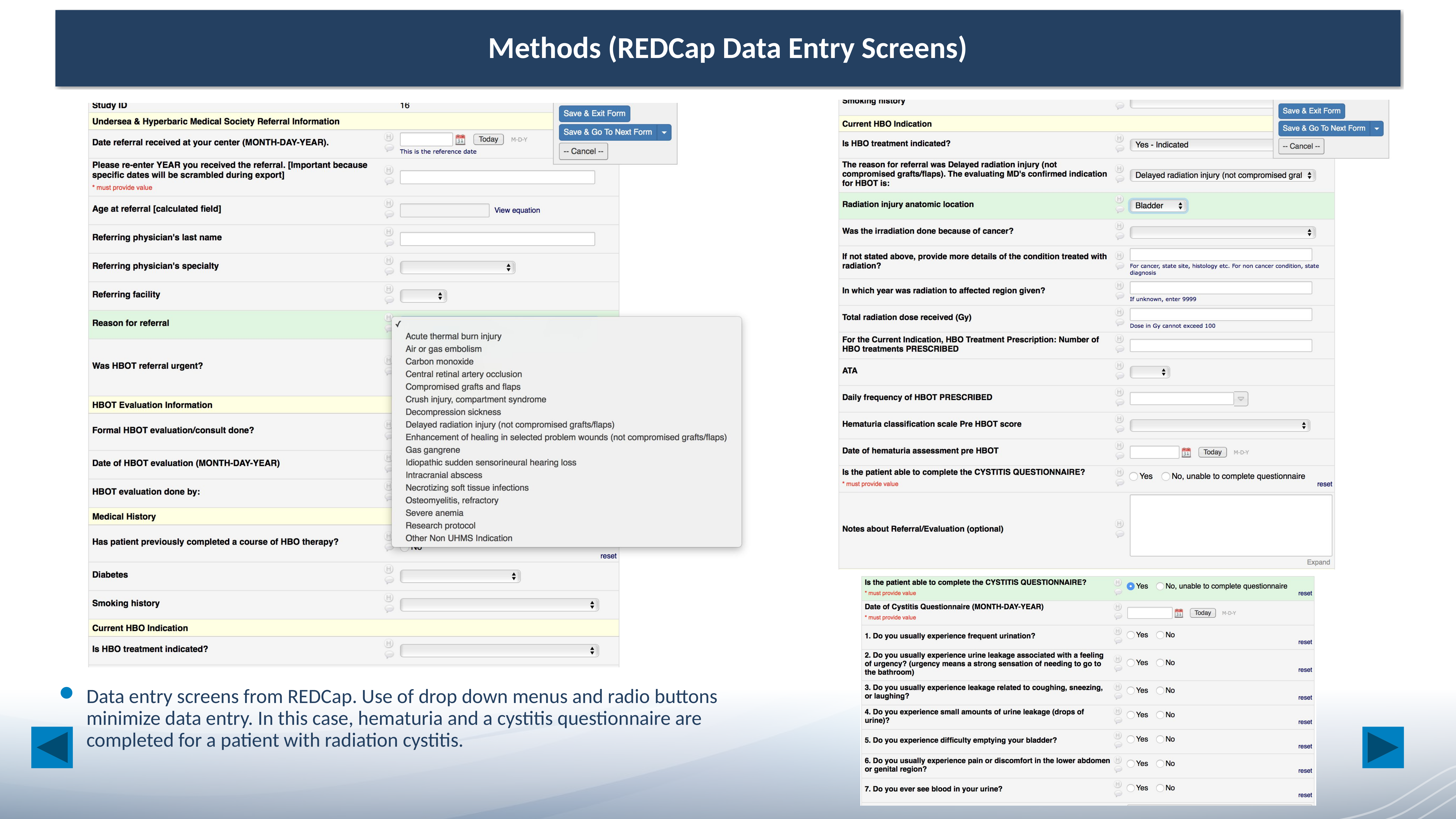

Methods (REDCap Data Entry Screens)
Data entry screens from REDCap. Use of drop down menus and radio buttons minimize data entry. In this case, hematuria and a cystitis questionnaire are completed for a patient with radiation cystitis.

## Slide 3
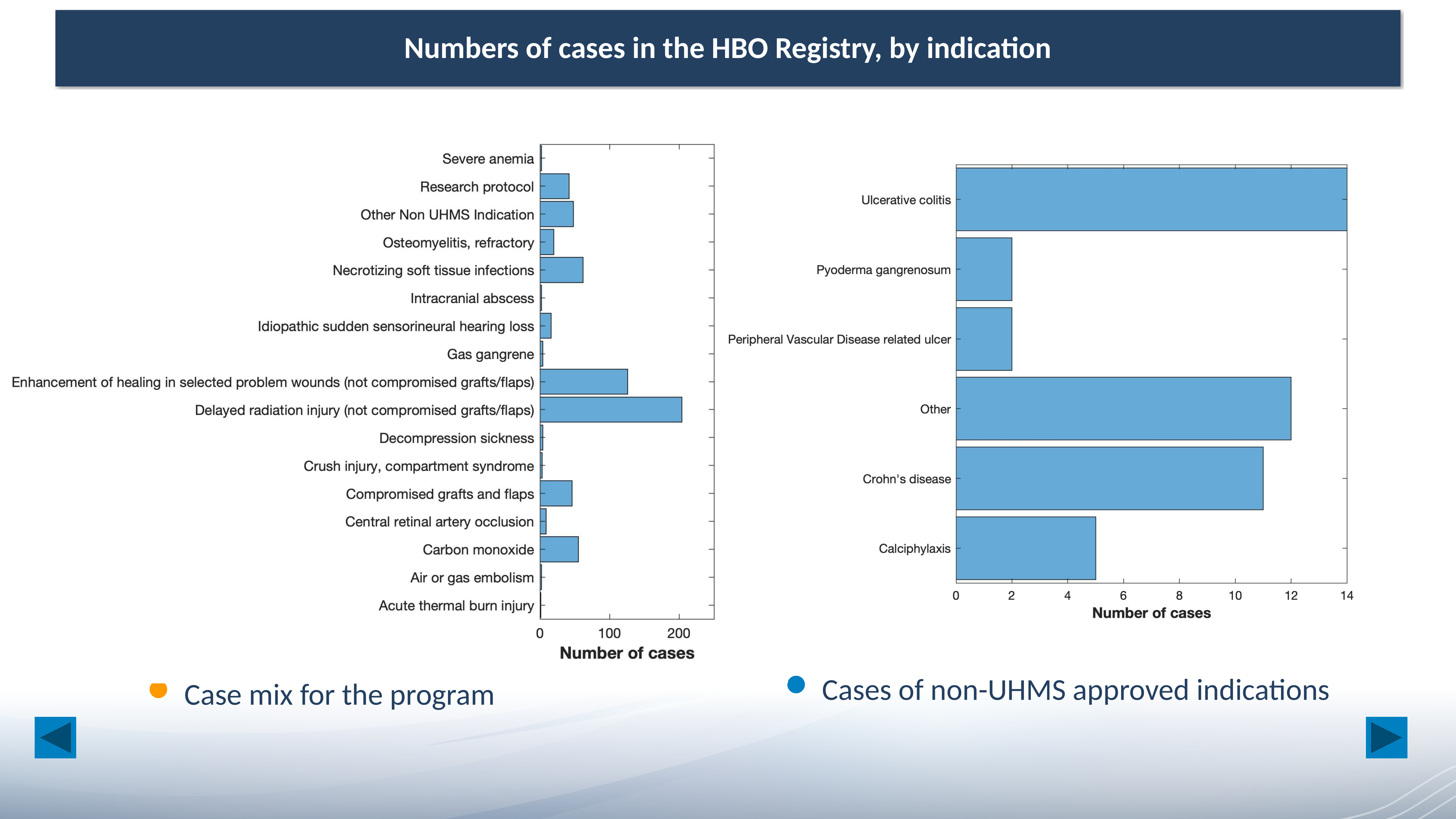

Numbers of cases in the HBO Registry, by indication
 Cases of non-UHMS approved indications
 Case mix for the program

## Slide 4
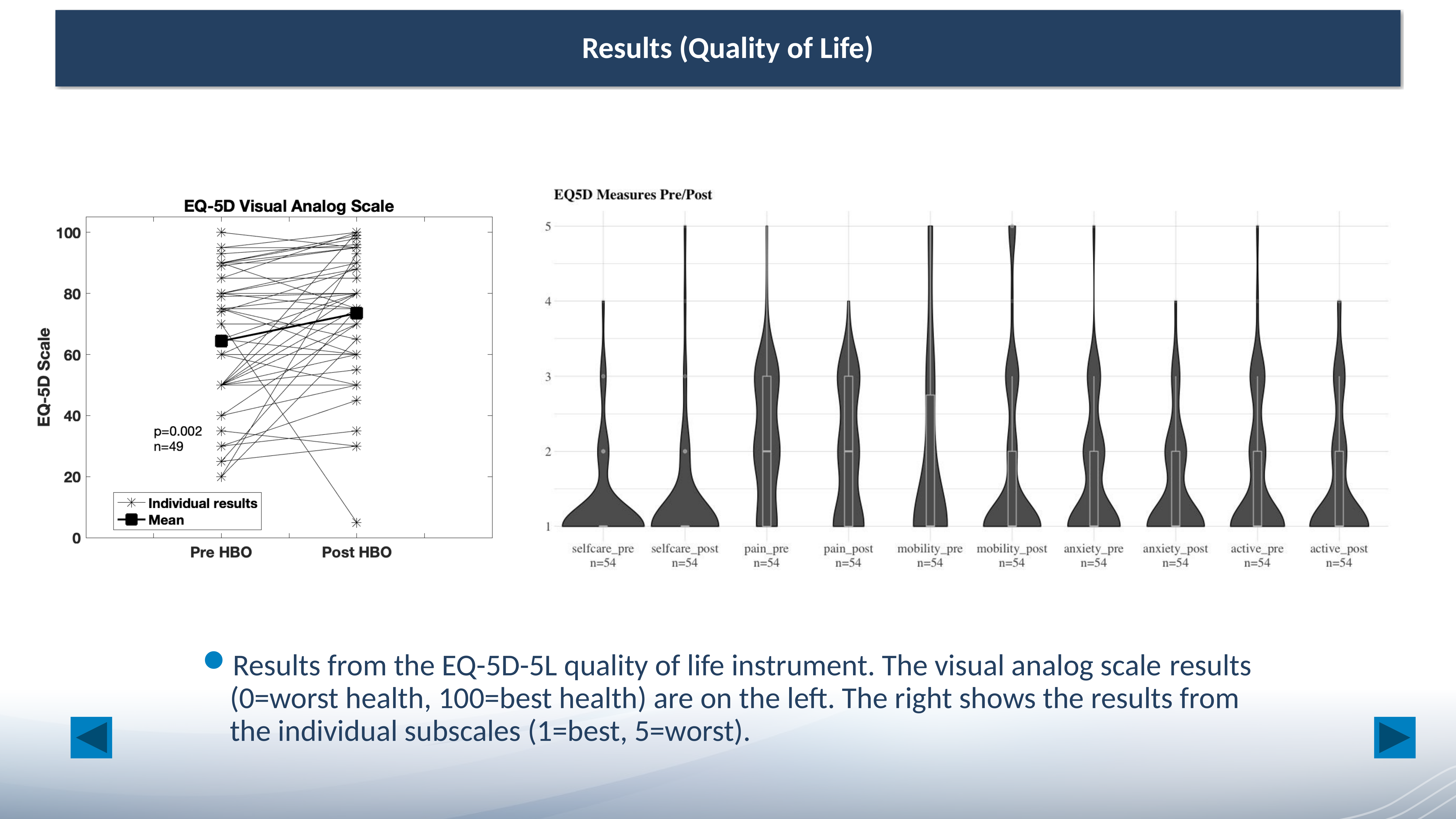

Results (Quality of Life)
Results from the EQ-5D-5L quality of life instrument. The visual analog scale results (0=worst health, 100=best health) are on the left. The right shows the results from the individual subscales (1=best, 5=worst).

## Slide 5
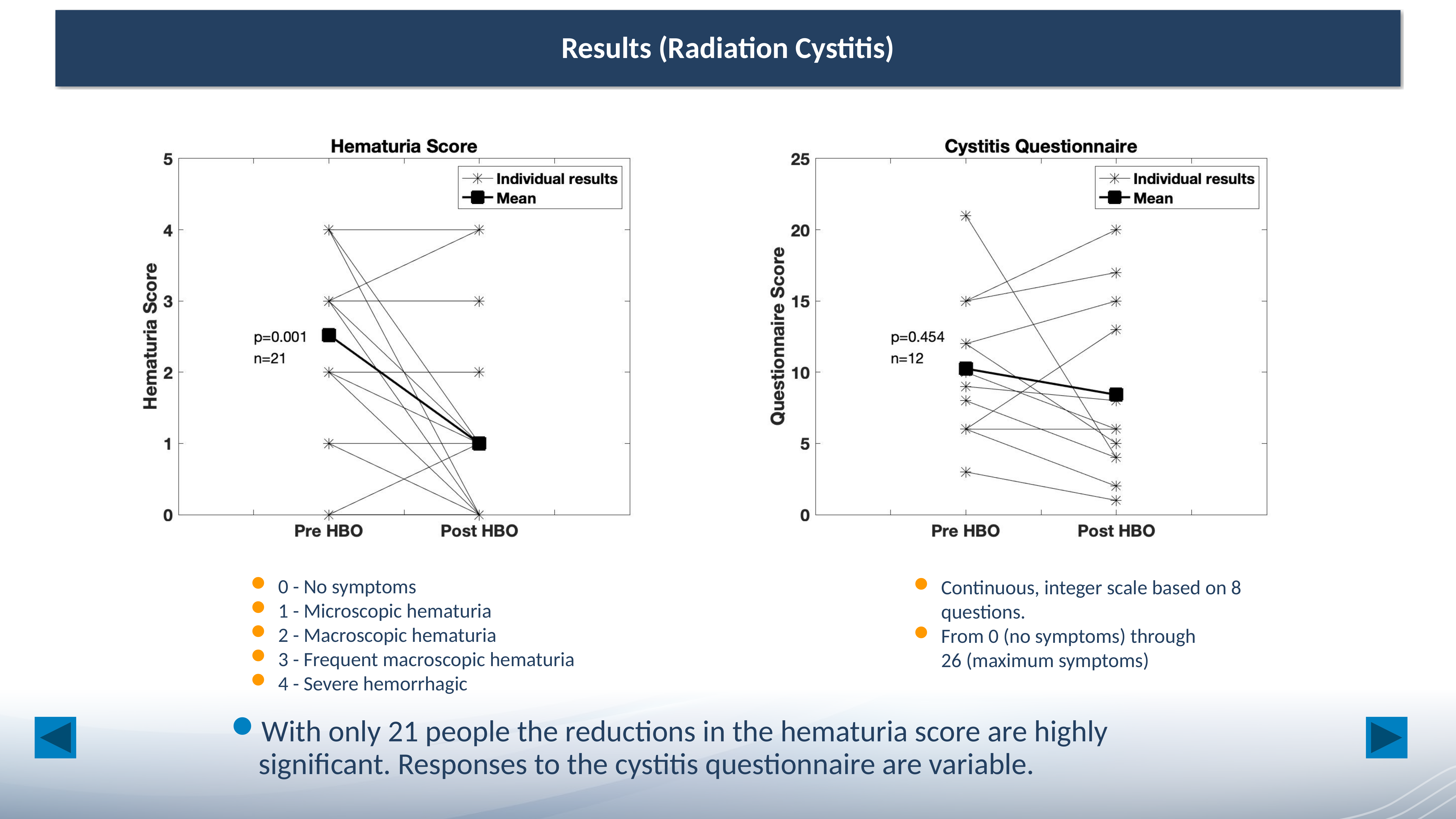

Results (Radiation Cystitis)
0 - No symptoms
1 - Microscopic hematuria
2 - Macroscopic hematuria
3 - Frequent macroscopic hematuria
4 - Severe hemorrhagic
Continuous, integer scale based on 8 questions.
From 0 (no symptoms) through 26 (maximum symptoms)
With only 21 people the reductions in the hematuria score are highly significant. Responses to the cystitis questionnaire are variable.

## Slide 6
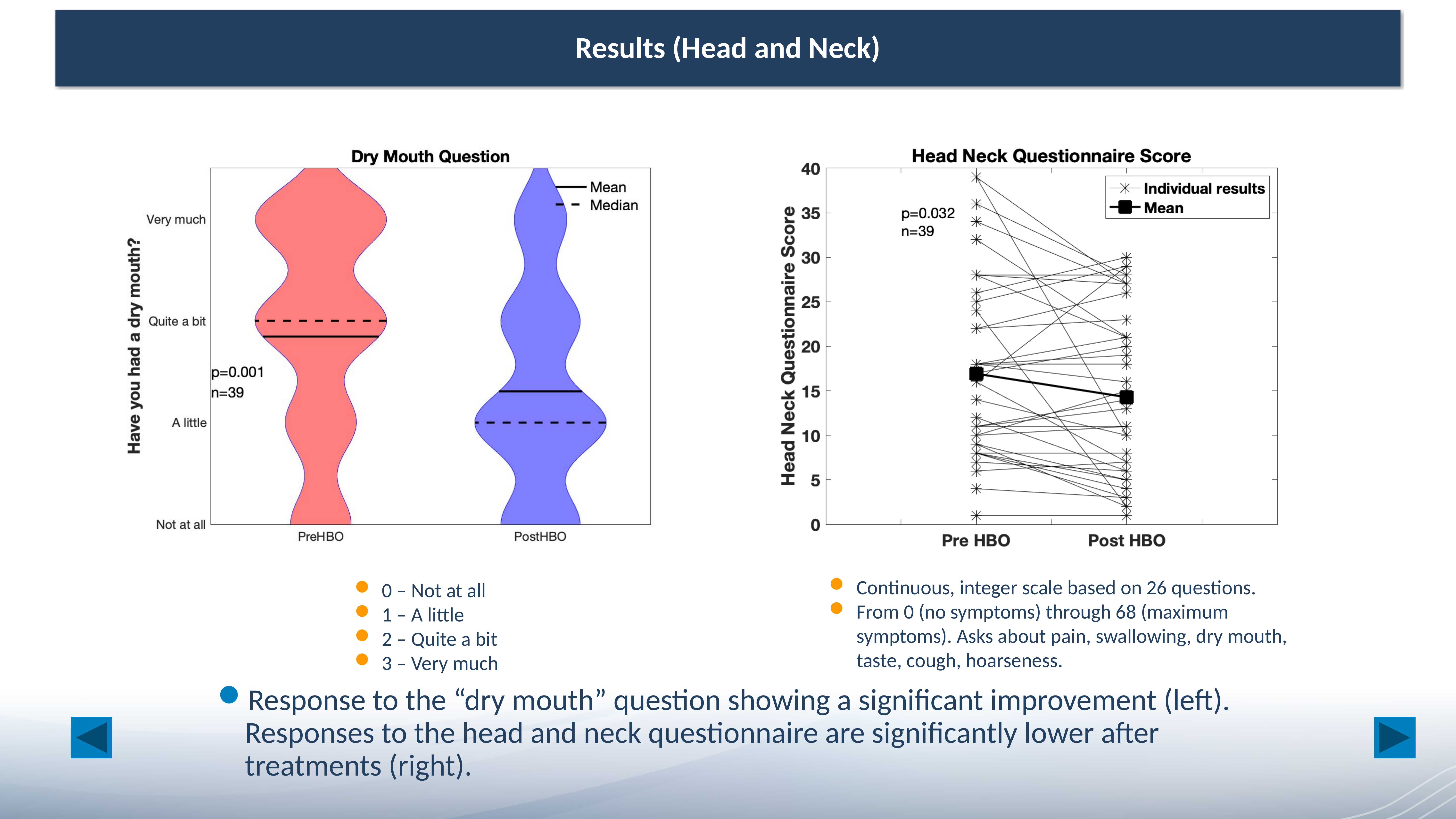

Results (Head and Neck)
Continuous, integer scale based on 26 questions.
From 0 (no symptoms) through 68 (maximum symptoms). Asks about pain, swallowing, dry mouth, taste, cough, hoarseness.
0 – Not at all
1 – A little
2 – Quite a bit
3 – Very much
Response to the “dry mouth” question showing a significant improvement (left). Responses to the head and neck questionnaire are significantly lower after treatments (right).

## Slide 7
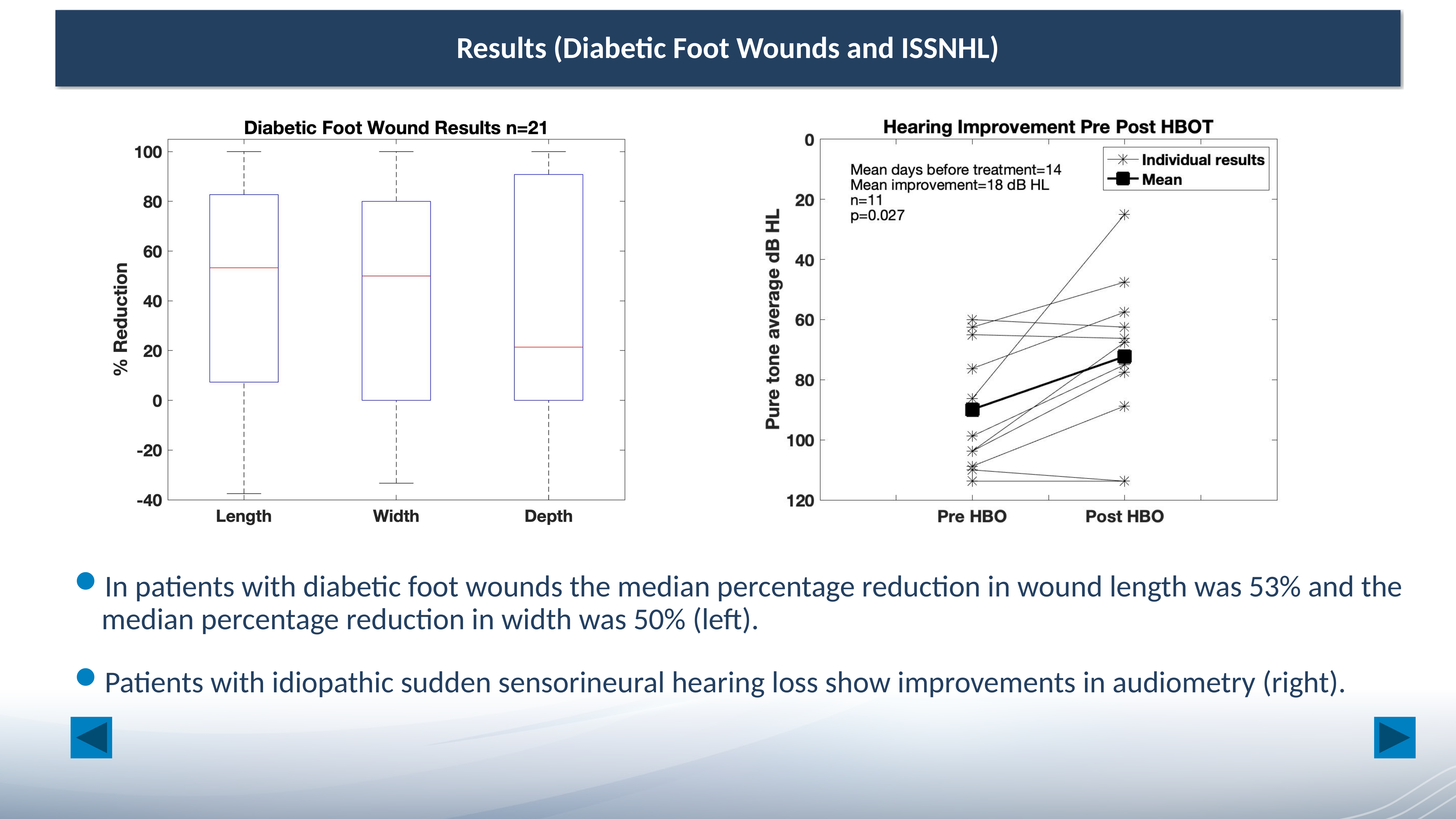

Results (Diabetic Foot Wounds and ISSNHL)
In patients with diabetic foot wounds the median percentage reduction in wound length was 53% and the median percentage reduction in width was 50% (left).
Patients with idiopathic sudden sensorineural hearing loss show improvements in audiometry (right).

## Slide 8
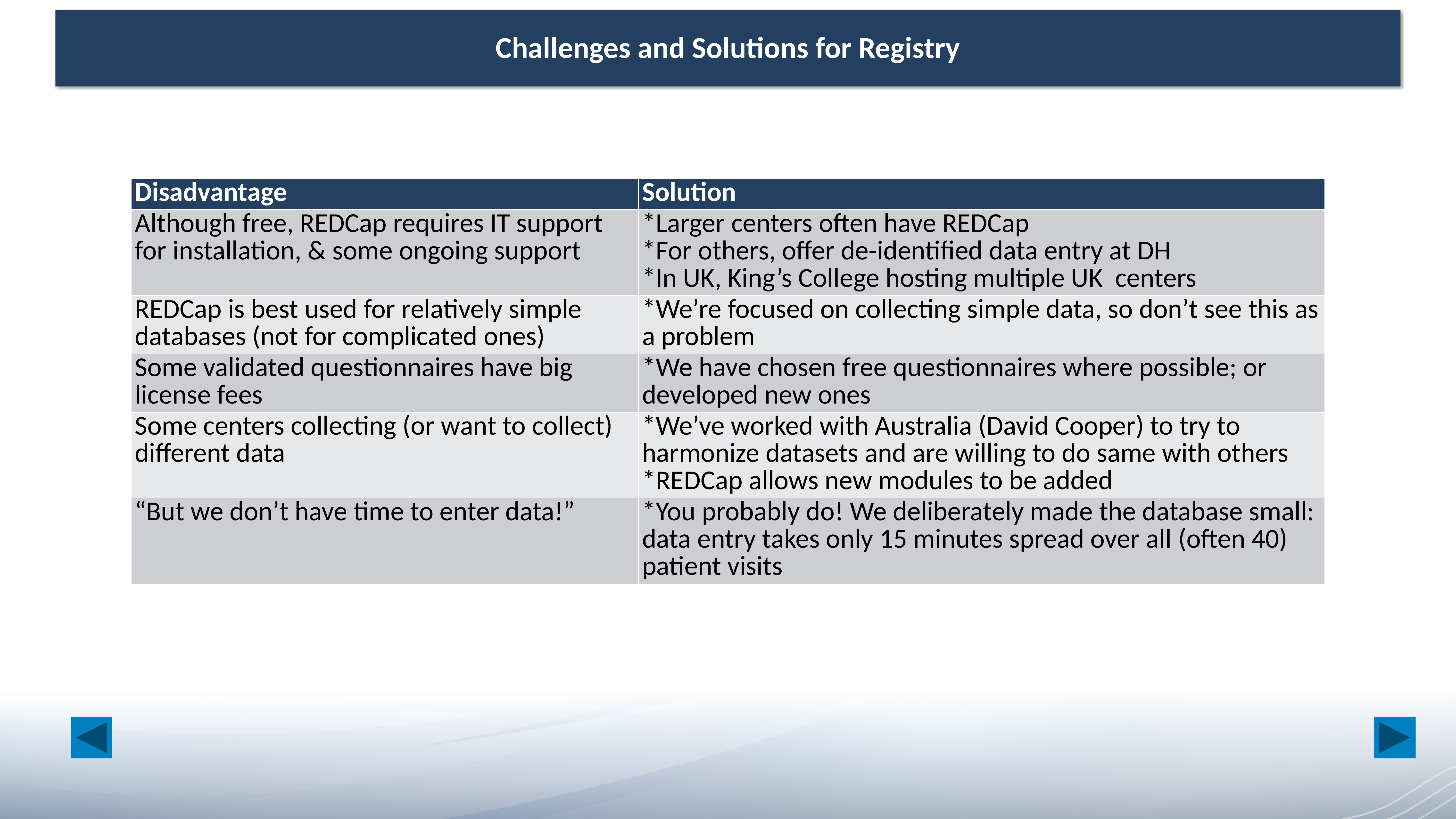

Challenges and Solutions for Registry
| Disadvantage | Solution |
| --- | --- |
| Although free, REDCap requires IT support for installation, & some ongoing support | \*Larger centers often have REDCap \*For others, offer de-identified data entry at DH \*In UK, King’s College hosting multiple UK centers |
| REDCap is best used for relatively simple databases (not for complicated ones) | \*We’re focused on collecting simple data, so don’t see this as a problem |
| Some validated questionnaires have big license fees | \*We have chosen free questionnaires where possible; or developed new ones |
| Some centers collecting (or want to collect) different data | \*We’ve worked with Australia (David Cooper) to try to harmonize datasets and are willing to do same with others \*REDCap allows new modules to be added |
| “But we don’t have time to enter data!” | \*You probably do! We deliberately made the database small: data entry takes only 15 minutes spread over all (often 40) patient visits |

## Slide 9
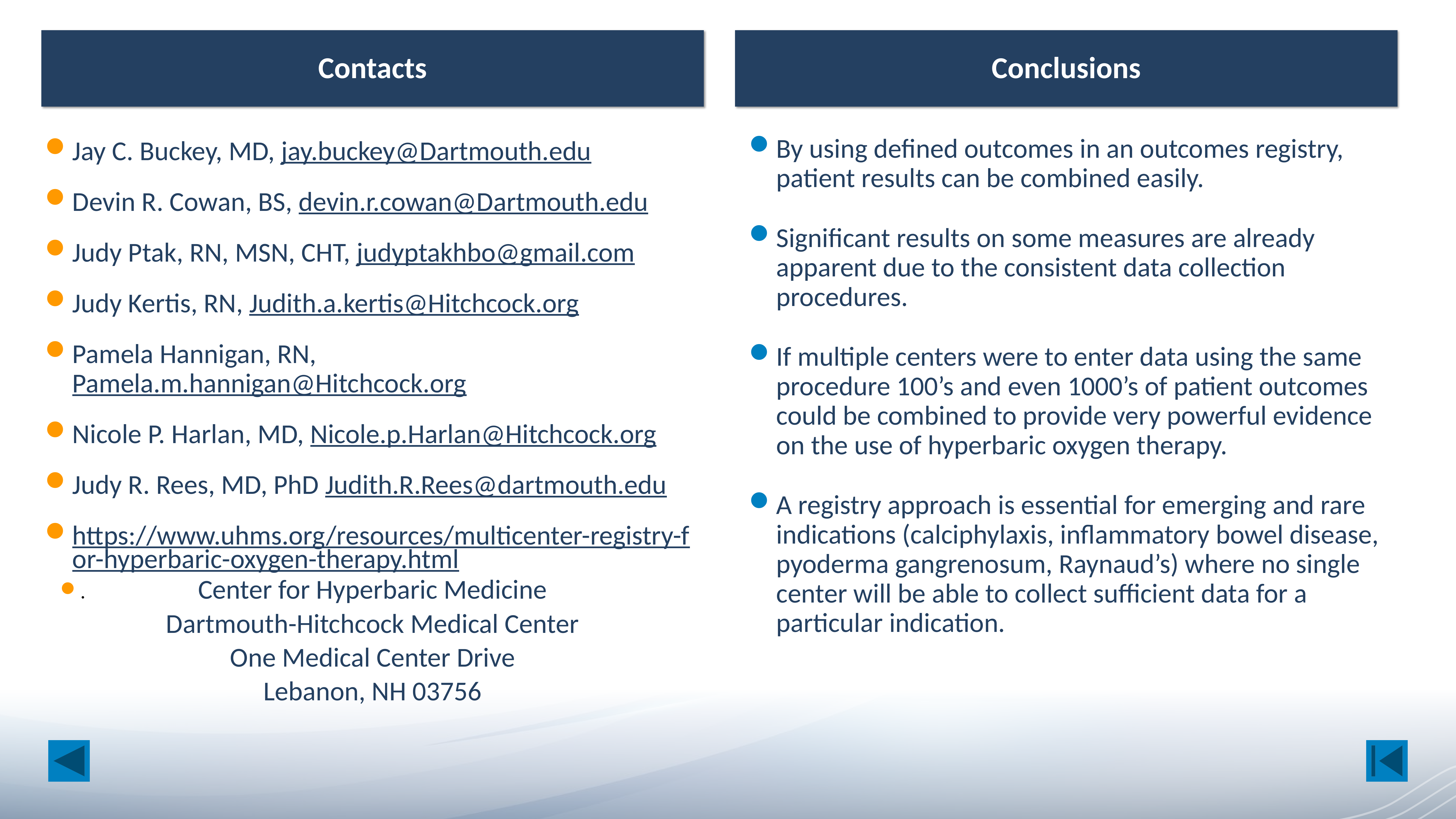

Contacts
Conclusions
By using defined outcomes in an outcomes registry, patient results can be combined easily.
Significant results on some measures are already apparent due to the consistent data collection procedures.
If multiple centers were to enter data using the same procedure 100’s and even 1000’s of patient outcomes could be combined to provide very powerful evidence on the use of hyperbaric oxygen therapy.
A registry approach is essential for emerging and rare indications (calciphylaxis, inflammatory bowel disease, pyoderma gangrenosum, Raynaud’s) where no single center will be able to collect sufficient data for a particular indication.
Jay C. Buckey, MD, jay.buckey@Dartmouth.edu
Devin R. Cowan, BS, devin.r.cowan@Dartmouth.edu
Judy Ptak, RN, MSN, CHT, judyptakhbo@gmail.com
Judy Kertis, RN, Judith.a.kertis@Hitchcock.org
Pamela Hannigan, RN, Pamela.m.hannigan@Hitchcock.org
Nicole P. Harlan, MD, Nicole.p.Harlan@Hitchcock.org
Judy R. Rees, MD, PhD Judith.R.Rees@dartmouth.edu
https://www.uhms.org/resources/multicenter-registry-for-hyperbaric-oxygen-therapy.html
Center for Hyperbaric Medicine
Dartmouth-Hitchcock Medical Center
One Medical Center Drive
Lebanon, NH 03756
.
